# Attention-guided cascaded network with pixel-importance-balance loss for retinal vessel segmentation

**DOI:** 10.3389/fcell.2023.1196191

**Published:** 2023-05-09

**Authors:** Hexing Su, Le Gao, Yichao Lu, Han Jing, Jin Hong, Li Huang, Zequn Chen

**Affiliations:** ^1^ Faculty of Intelligent Manufacturing, Wu Yi University, Jiangmen, China; ^2^ Guangdong Provincial Key Laboratory of South China Structural Heart Disease, Guangdong Provincial People’s Hospital (Guangdong Academy of Medical Sciences), Southern Medical University, Guangzhou, China; ^3^ Medical Research Institute, Guangdong Provincial People’s Hospital (Guangdong Academy of Medical Sciences), Southern Medical University, Guangzhou, China; ^4^ Faculty of Social Sciences, Lingnan University, Hongkong, China

**Keywords:** retinal vessel segmentation, deep learning, attention mechanism, U-net, pixel-wise loss

## Abstract

Accurate retinal vessel segmentation from fundus images is essential for eye disease diagnosis. Many deep learning methods have shown great performance in this task but still struggle with limited annotated data. To alleviate this issue, we propose an Attention-Guided Cascaded Network (AGC-Net) that learns more valuable vessel features from a few fundus images. Attention-guided cascaded network consists of two stages: the coarse stage produces a rough vessel prediction map from the fundus image, and the fine stage refines the missing vessel details from this map. In attention-guided cascaded network, we incorporate an inter-stage attention module (ISAM) to cascade the backbone of these two stages, which helps the fine stage focus on vessel regions for better refinement. We also propose Pixel-Importance-Balance Loss (PIB Loss) to train the model, which avoids gradient domination by non-vascular pixels during backpropagation. We evaluate our methods on two mainstream fundus image datasets (i.e., DRIVE and CHASE-DB1) and achieve AUCs of 0.9882 and 0.9914, respectively. Experimental results show that our method outperforms other state-of-the-art methods in performance.

## 1 Introduction

Retinal vessel analysis is a non-invasive and cost-effective test that ophthalmologists and other specialists routinely use (Chatziralli et al., 2012; Ji et al., 2023). Physicians can diagnose and track many diseases (e.g., macular degeneration, hypertension, diabetes) by looking at morphologic information related to retinal vessels (e.g., curvature, length, and width) because these diseases cause morphologic changes in the retinal vessels (Olafsdottir et al., 2011). The segmentation of retinal vessels is an essential foundation for the quantitative analysis of fundus images. Since manual segmentation is time-consuming, labor-intensive, and relies on professionals’ subjective judgment, many researchers have turned to computer-aided intervention to achieve automatic retinal vessel segmentation (Zhao et al., 2022a; Zhao et al., 2022b).

**FIGURE 1:**
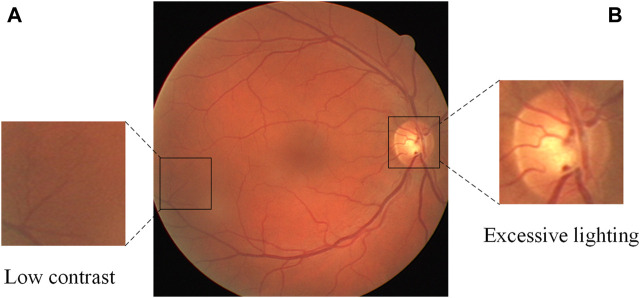
A fundus retinal image from the DRIVE database, containing thin blood vessels with low contrast **(A)** and over-illuminated optic disc **(B)**.

Automatic retinal vessel segmentation is an important research problem in the field of computer vision, and its main purpose is to separate vascular and non-vascular regions from fundus images. Solving this problem is of great significance for clinical diagnosis and research in the field of ophthalmology. Because it can promote the early detection and treatment of eye diseases, and provide clinicians with a fast, accurate, and reliable analysis method. However, due to the complexity and variability of fundus images, finding every vessel without introducing too many false positives is difficult, especially for thin vessels. When improper imaging illumination, sensor noise, and other factors are considered, things become even more complicated because vital vessel information may be lost as a result. In Figure 1, for example, there is usually over-illumination near the optic disc, causing some vessels near the optic disc to lose feature information. Thin vessels are typically found in darker, lower contrast areas, and their width is only one or a few pixels when compared to thick vessels, so they are easily overlooked. To address these challenges, many methods for automatic retinal vessel segmentation have been proposed in the past few decades. For example, the blood vessel tracking method (Yin et al., 2012; Tolias and Panas, 1998; Chutatape et al., 1998) begins by selecting a starting point in the fundus image and utilizes a specific tracking strategy to progressively extend along the blood vessel path, culminating in a comprehensive segmentation of the blood vessels. The method based on morphology (Sazak et al., 2019; (Zana and Klein, 2001) performs some morphological operator processing (such as erosion, dilation, opening and closing operations, etc.) on the fundus image to realize the segmentation of blood vessels. In addition, methods based on traditional machine learning (Ricci and Perfetti, 2007; Staal et al., 2004; Lupascu et al., 2010) manually extract vascular features (such as shape, texture, etc.), and send these features to classifiers (such as support vector machines, decision trees, etc.) for training to achieve segmentation. Although these traditional retinal vessel segmentation methods have certain advantages and applicability, there are still limitations in the processing of fundus image noise, generalization, etc.

Due to the powerful feature extraction ability of the convolutional neural network, it has gradually become the mainstream method for segmentation tasks (Khandouzi et al., 2022). Fully convolutional network (Long et al., 2015) is a pioneering work using a convolutional neural network in image segmentation. It discards the fully connected layers of the Very deep convolutional networks (Simonyan and Zisserman, 2014), and the entire network uses convolution operations for feature extraction, followed by upsampling of the feature maps to restore the original resolution. However, FCN is not sensitive to the details of objects in the image, resulting in the loss of edge details of many objects. Subsequently, based on the idea of an encoder-decoder structure, Ronneberger et al. (2015) proposed U-Net, which made up for the lack of details of FCN to a certain extent by using skip connection operation, and gradually became the mainstream model in the field of medical image segmentation. In recent years, many U-Net based variants (Jin et al., 2019; Guo et al., 2021a; Alom et al., 2019; Guo et al., 2021b; Wang et al., 2020b; Wu et al., 2021; Zhang et al., 2019) for the task of retinal vessel segmentation have emerged, but they suffer from insufficient vessel information and features due to the limited number of fundus images with dense annotations in the public dataset [e.g., DRIVE (Staal et al., 2004), CHASE_DB1 (Owen et al., 2009)]. In this case, some studies (Wang et al., 2020a; Xia et al., 2018; Li et al., 2020) have shown that the coarse-to-fine segmentation architecture is beneficial for extracting more vascular information from limited fundus images. However, these works simply transfer vessel feature maps (such as concatenation or addition) between coarse and fine stages, which makes the fine stage unable to align vessel regions for better refinement and leads to suboptimal performance. To address this problem, we propose an Attention-Guided Cascaded Network (AGC-Net), which can learn more valuable vascular information from limited retinal fundus images. AGC-Net consists of two identical U-shaped backbones for coarse and fine representation learning. Specifically, the coarse-stage backbone generates a rough vessel probability map from the fundus image. In contrast, the fine-stage backbone acts as a post-processing module to further refine missing vessel details from this map. This coarse-to-fine representation learning can allow those misclassified pixels to be corrected, especially those blood vessel pixels whose predicted probability value is slightly lower than the segmentation threshold (usually taken as 0.5). Then, we incorporate an inter-stage attention module (ISAM) to cascade the two-stage backbone in AGC-Net. ISAM uses a multi-scale spatial attention mechanism to promote fine-stage backbone focus on vessel regions for better refinement.

Furthermore, deep learning-based segmentation models are typically trained using pixel-wise loss (e.g., Cross Entropy Loss). It creates a loss by comparing the per-pixel difference between the vessel probability map generated by the segmentation model and the Ground Truths labeled by human experts and then uses that loss for gradient computation and backpropagation. In the pixel-wise loss, each pixel is treated with equal importance (i.e., the loss weights are all 1.0) and the loss is calculated separately for each pixel. However, when the ratio of background pixels and blood vessel pixels in the retinal image is seriously unbalanced (the ratio is about 8:2), pixel-wise loss makes the optimization of the segmentation results severely affected by the background, which leads to inaccurate blood vessel segmentation. To prevent the gradient from being dominated by many background pixels during backpropagation, we propose a Pixel-Importance-Balance Loss (PIB Loss) for training the blood vessel segmentation model. It scales the loss weights for each pixel according to the number of vessels around them. Our primary contributions are as follows:

1. We propose AGC-Net, a deep learning-based segmentation model for retinal vessel segmentation that aims to improve segmentation results from limited fundus data by allowing misclassified pixels to be corrected.
2. We propose ISAM to cascade two backbones in AGC-Net, which intends to enable the fine-stage backbone to focus more effectively on vascular regions for better refinement.
3. We propose PIB Loss for training vessel segmentation model, which can prevent the gradient from being dominated by many background pixels during backpropagation.

The remainder of this paper is structured as follows. Section 2 reviews the studies related to retinal image vessel segmentation. Section 3 describes our method. Data and experimental details are described in Section 4. Section 5 evaluates our approach quantitatively and qualitatively and presents experimental results. Finally, in Section 6, we conclude.

## 2 Related works

In the past decades, many automatic retinal segmentation algorithms have been proposed, and they can be broadly classified into three categories.

The first class of algorithms is designed using traditional computer vision methods for vessel segmentation and is based on the inherent morphological prior knowledge of retinal vessels. For example, threshold-based methods (Roychowdhury et al., 2015; Zardadi et al., 2016), filter-based methods (Mendonca and Campilho, 2006; Fraz et al., 2012a; Zhang et al., 2015) and vessel tracking-based methods (Nayebifar and Moghaddam, 2013; Vázquez et al., 2013). Roychowdhury et al. (2015) designed an iterative adaptive thresholding method to improve the robustness of vessel segmentation. Oliveira et al. (2016) enhanced the vessels by combining three filters: the matched filter, the Gabor Wavelet filter, and Frangi’s filter. Zhang et al. (2010) detected blood vessels by thresholding the response of the retinal image to the matched filter and later adjusted the threshold by the image’s response to the first-order derivative of Gaussian. Nayebifar and Moghaddam (2013) used least-cost matching, global graph optimization, and Dijkstra’s algorithm to track vessels as a way to ensure vessel continuity. Traditional algorithms based on morphological priors are relatively simple in principle, but they are unsupervised methods that lack label constraints with annotations and produce less accurate vessel segmentation results.

The second class of algorithms is based on traditional machine learning approaches, identifying blood vessel pixels by feeding manually designed features to a trained classifier. Staal et al. (2004) created feature vectors from blood vessel centerlines and then classified them using a k-nearest neighbor classifier. Simple feature vectors were created based on the texture, local intensity, spatial properties, and geometry of blood vessels, and some researchers (Fraz et al., 2012b; Memari et al., 2017; Lupascu et al., 2010) tried to use ensemble learning methods (e.g., Bagging and Boosting) to classify blood vessel pixels. Ricci et al. (Staal et al., 2004) used linear detectors and support vector machines to complete the segmentation representation of blood vessels. The performance of traditional machine learning-based methods is heavily influenced by manually designed features. However, these features are typically defined empirically, resulting in bias and poor generalization performance.

The third class of algorithms is the deep learning-based approach, which automatically extracts blood vessel features rather than manually designed features through powerful convolutional neural networks. U-Net (Ronneberger et al., 2015) has become the most widely used model in the medical field of image segmentation, and several U-Net variants have made significant progress in retinal vessel segmentation. Alom et al. (2019) used the idea of recurrent neural networks and proposed a recurrent convolution in U- Net instead of a normal convolution to effectively accumulate more vessel features. Jin et al. (2019) integrated deformable convolution into U-Net. This convolution operation can adaptively adjust the receptive field according to the scale and shape of blood vessels to better capture various retinal blood vessels. SA-UNet (Guo et al., 2021b) and CAR-UNet (Guo et al., 2021a), proposed by Guo et al., respectively introduce attention mechanisms of spatial dimension and channel dimension in U-Net to improve the vessel segmentation performance of U-Net. IterNet (Li et al., 2020) and CTF-Net (Wang et al., 2020a) have shown that vessel segmentation performance can be improved based on cascades using multiple U-Nets, and we will implement a similar strategy in our method.

## 3 Methodology

**FIGURE 2:**
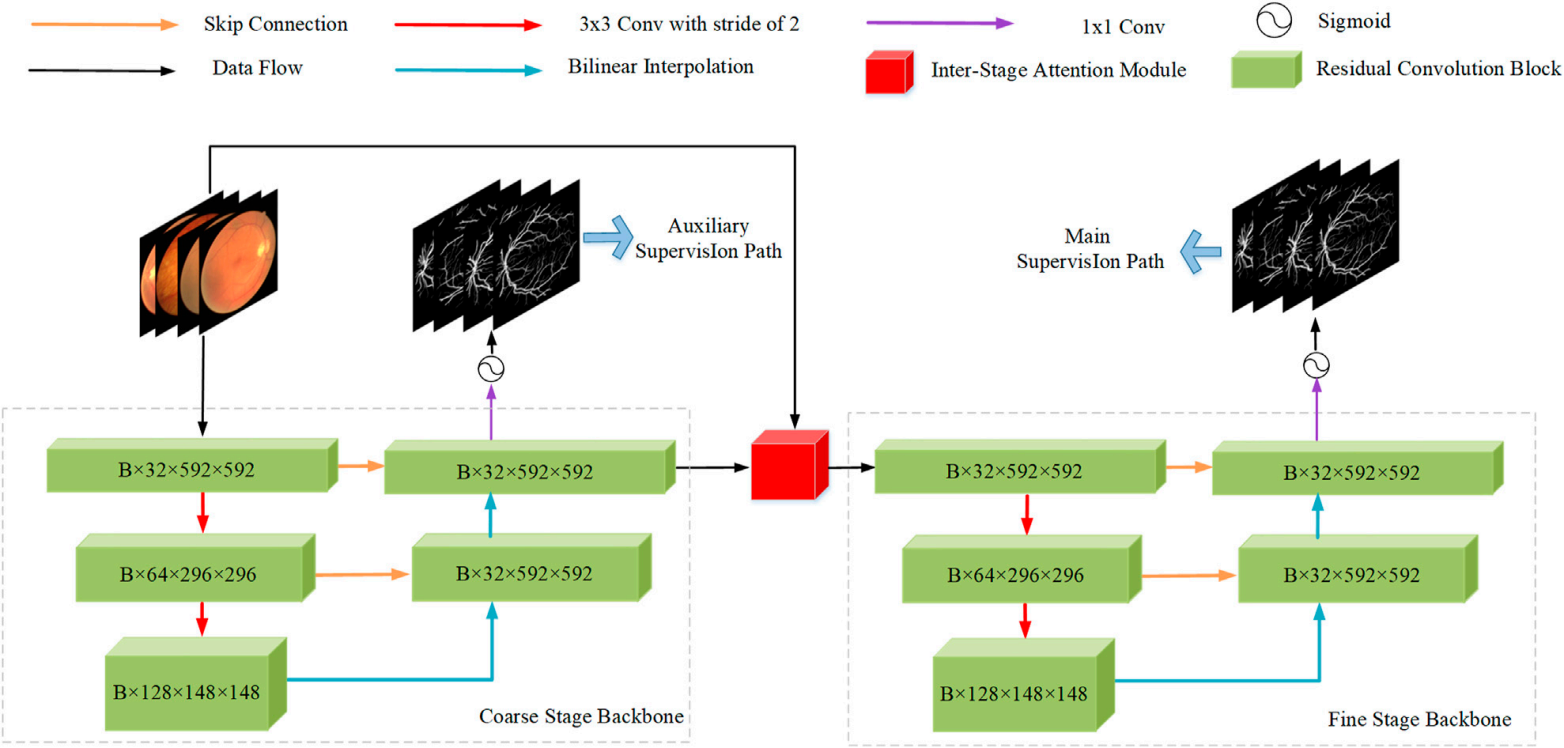
Network architecture of the proposed AGC-Net.

This study aims to accurately segment retinal vessels in fundus images using deep learning methods. Inspired by IterNet (Li et al., 2020) and CBAM (Woo et al., 2018), we propose our model AGC-Net by combining their advantages. As shown in Figure 2, the model is implemented based on a U-shape architecture and consists of three main ideas: residual convolution block, inter-stage attention module, and cascaded refinement structure design. In addition, we also propose PIB Loss for model training. We detail the proposed model and loss function below.

### 3.1 Network architecture

Figure 2 **s**hows our proposed AGC-Net vessel segmentation model. The model consists of a representation learning cascade of coarse and fine stages and aims to use the fine stage as a post-processing module to give pixels misclassified by the coarse stage a chance to relearn. Specifically, first, the fundus image passes through the backbone of the coarse stage to generate a rough vessel prediction probability map as an intermediate output. Then, ISAM (See Figure 4) uses a multi-scale attention mechanism on this intermediate output to generate feature maps of enhanced vessel regions. Finally, feature maps of enhanced vessel regions and fundus images are concatenated as the input of the fine-stage backbone to generate the final refined vessel segmentation map.

**FIGURE 3:**
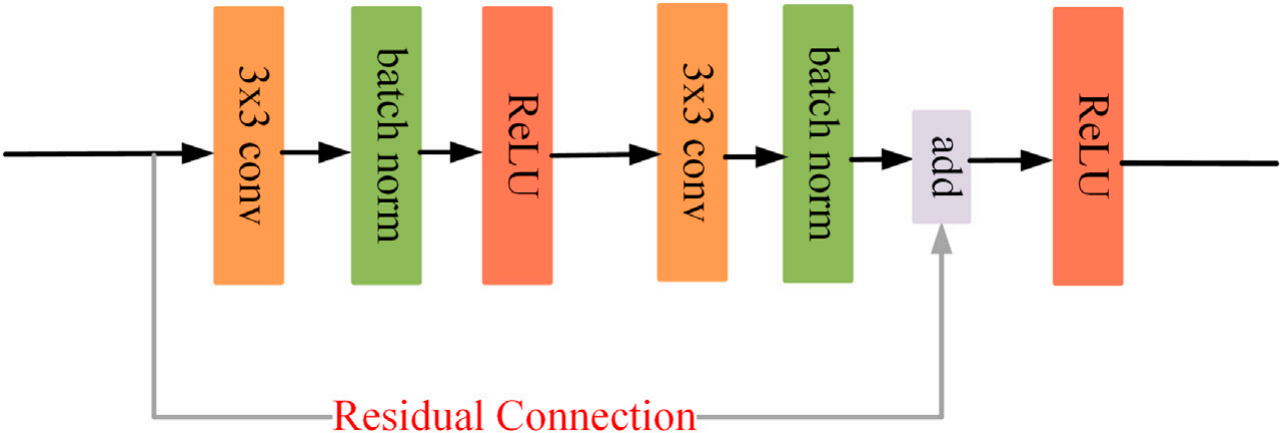
Residual convolution block.

Both stages are equipped with a U-shaped backbone for their respective learning tasks. The U-backbone is an encoder and decoder structure that generates multi-scale vessel feature maps to identify vessels of different lengths. Specifically, the encoder extracts the vascular features of the fundus image through a residual convolution block (See Figure 3). Each block includes a convolutional layer, a batch normalization layer, and a ReLU activation layer, and we use residual connections to speed up the convergence of the model. To obtain a larger receptive field, downsampling is necessary. This operation is implemented by a convolution with stride 2. At each downsampling stage, the size of the feature map is halved, and the number of channels is doubled. Since too much downsampling will lose the spatial information of vessels, there are only two downsampling stages in the backbone, each with 32, 64, and 128 channels. In the decoder part, we upsample the vessel feature map by bilinear interpolation and compensate for the lost spatial information of the vessel by skip connections to receive the feature map of the encoder. Finally, through a 1 × 1 convolution and a Sigmoid layer, we get the final vessel segmentation.

Since AGC-Net is composed of two backbone network cascades, it may suffer from the gradient disappearance problem due to the increase in network depth. In response to this problem, inspired by DSN (Lee et al., 2015), in addition to adding the main supervision path to the network’s final output, we also add an auxiliary supervision path to the intermediate output of the backbone in the coarse stage. During training, the loss functions of these two supervised paths are weighted into the overall loss function, which helps gradient backpropagation back to shallower layers and speeds up model convergence. Specifically, we compare the predicted probability maps outputted from the backbone of the two stages with the ground truth and compute the loss for backpropagation using the PIB loss (see Section 5.3), as shown in the following figure:

$$Loss={Loss}_{main}+\gamma {Loss}_{auxl}$$

$${Loss}_{main}=PIB\left({PM}_{f},GT\right)$$

$${Loss}_{auxl}=PIB\left({PM}_{c},GT\right)$$

where ${Loss}_{main}$ and ${Loss}_{auxl}$ are the losses generated by the backbone of the fine stage and coarse stage, respectively, and the weight *λ* represents the trade-off between the two losses, which we set as 1.0 in the experiments. PIB represents the proposed PIB loss, and ${PM}_{f}$ , ${PM}_{c}$ and GT are respectively the predicted probability map of the coarse stage, the predicted probability map of the fine stage, and the ground truths.

### 3.2 Inter-stage attention module

ISAM is proposed to enhance the vessel region of the intermediate output, which can facilitate the fine-stage backbone to focus on vessel regions for better refinement.

**FIGURE 4:**
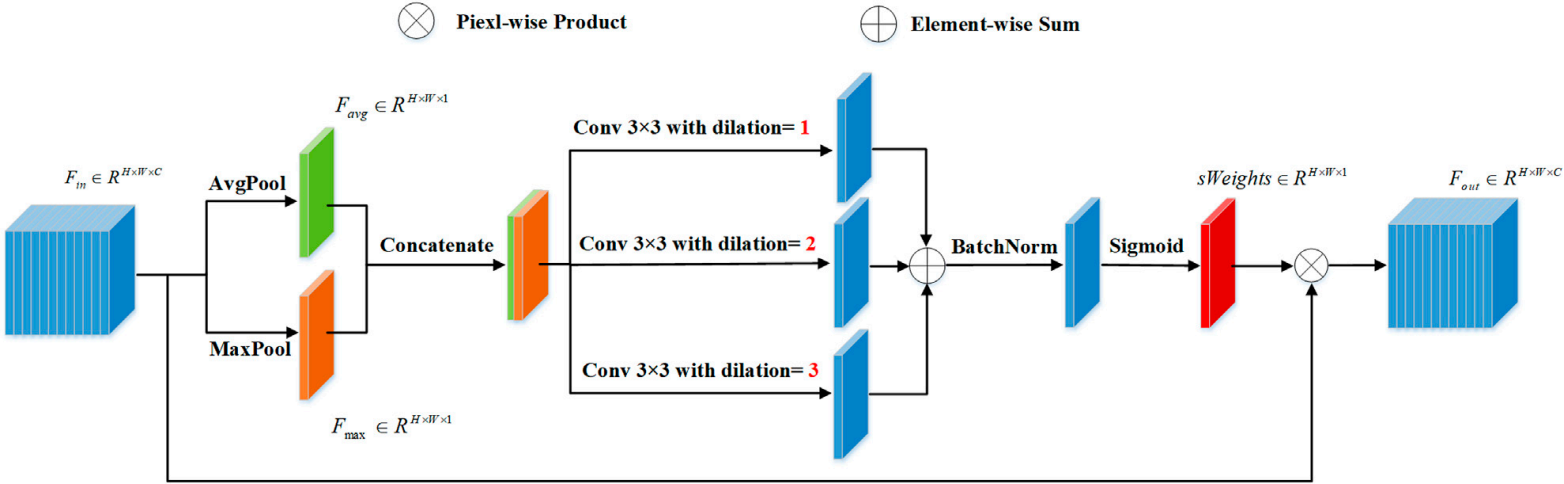
Diagram of the proposed Inter-Stage Attention Module (ISAM).

As shown in Figure 4, we assume that the ISAM has an input resolution of $F_{in}\in R^{H\times W\times C}$ , we first apply average and maximum pooling operations to $F_{in}$ along the channel axis to obtain spatial feature descriptors $F_{avg}\in R^{H\times W\times 1}$ and $F_{\max}\in R^{H\times W\times 1}$ , as shown in the following equation:

$$F_{avg}\in R^{H\times W\times 1}=avgPool\left(F_{in}\right)$$

$$F_{\max}\in R^{H\times W\times 1}=maxPool\left(F_{in}\right)$$

Subsequently, to enhance the vessel region of $F_{in}$ , we concatenate these two spatial feature descriptors and use 3 × 3 convolution kernels with different dilation rates to model the neighborhood relationship of pixels. We add the results of the modeling and then use the sigmoid operation to generate the attention map $sWeights\in R^{H\times W\times 1}$ , as shown below:

$$sWeights=\sigma \left(BN\left(\varphi_{3,rate=1}\left(\left[F_{avg};F_{\max}\right]\right)+\varphi_{3,rate=2}\left(\left[F_{avg};F_{\max}\right]\right)+\varphi_{3,rate=3}\left(\left[F_{avg};F_{\max}\right]\right)\right)\right)$$

Among them, σ represents Sigmoid activation, BN represents Batch Normalization, and $\varphi_{3,rate=N}$ represents the 3 × 3 convolution with a dilation rate of N. It is worth mentioning that in the above process, the purpose of using convolution operations with different dilation rates is to integrate multi-scale context information when calculating the importance of pixels, to better encode the emphasized or suppressed positions. Finally, we obtain the ISAM output $F_{out}\in R^{H\times W\times C}$ based on the obtained spatial attention map and scaled feature map $F_{in}$ , as shown in the following equation:

$$F_{out}=sWeights⊗F_{in}$$

where ⊗ denotes pixel-wise product.

### 3.3 Pixel-importance-balance loss

There are three types of pixels in fundus images: background, thick vessels, and thin vessels. Their proportions in the fundus image vary from high to low. To balance the contributions of these three types of pixels in loss computation, we scale their loss weights according to the number of vessel pixels in their neighborhood. Specifically, for background pixels, we think that only background pixels near blood vessels need to be emphasized, as this can force the model to keep the predicted blood vessel thickness consistent with the thickness in the ground truth. Therefore, the loss weights of background pixels should be proportional to the number of blood vessel pixels in their neighborhood. As for blood vessel pixels, if they belong to thick blood vessels, they will be surrounded by more blood vessel pixels in the fundus image, and fewer if they belong to thin blood vessels. Therefore, to balance the contributions of these two kinds of vessel pixels, the loss weights of vessel pixels should be inversely proportional to the number of vessel pixels in their neighborhood.

**FIGURE 5:**
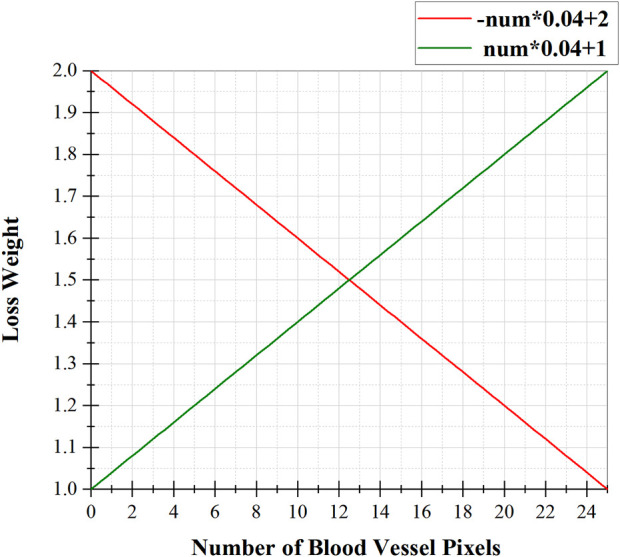
The relationship between the loss weight of a pixel and the number of blood vessel pixels around it, where the red function is used for vessel pixels and the green function is used for background pixels.

Algorithm 1 shows the calculation process of loss weights for different types of pixels in PIB loss. Firstly, the background pixels and vessel pixels of the Ground Truth are represented by 0 and 1. Secondly, the importance loss weight for each pixel is calculated as follows: with the pixel as the center, the number of pixels with value 1 (i.e., the number of surrounding vessel pixels) present in a box separated by 2 pixels is counted as ***num***. Thirdly, if the pixel belongs to a vessel, it is converted into a loss weight by an inverse proportional function -***num*** *0.04 + 2 (see Figure 5, red line), which emphasizes thin blood vessels; if the pixel belongs to the background, it is converted into loss weights by a direct proportional function ***num*** *0.04 + 1 (see Figure 5, green line), as shown below:

$$Loss\;weight\left(Y_{ij}\right)=\left\{\begin{array}{ll} -\left(\sum_{p=i-2}^{i+2}\sum_{q=j-2}^{j+2}Y_{pq}\right)*0.04+2 & if\;y_{ij}=1\\ \left(\sum_{p=i-2}^{i+2}\sum_{q=j-2}^{j+2}Y_{pq}\right)*0.04+1 & if\;y_{ij}=0 \end{array}\right.$$

Finally, the obtained loss weights are combined with the Cross Entropy, as shown below:

$$PIB\;Loss\left(P,Y\right)=\left\{\begin{array}{ll} -\sum weight\left(Y_{ij}\right)∙\log\left(P_{ij}\right) & if\;Y_{ij}=1\\ -\sum weight\left(Y_{ij}\right)∙\log\left(1-P_{ij}\right) & if\;Y_{ij}=0 \end{array}\right.$$

Algorithm 1. Loss weight calculation process of Our PIB Loss.
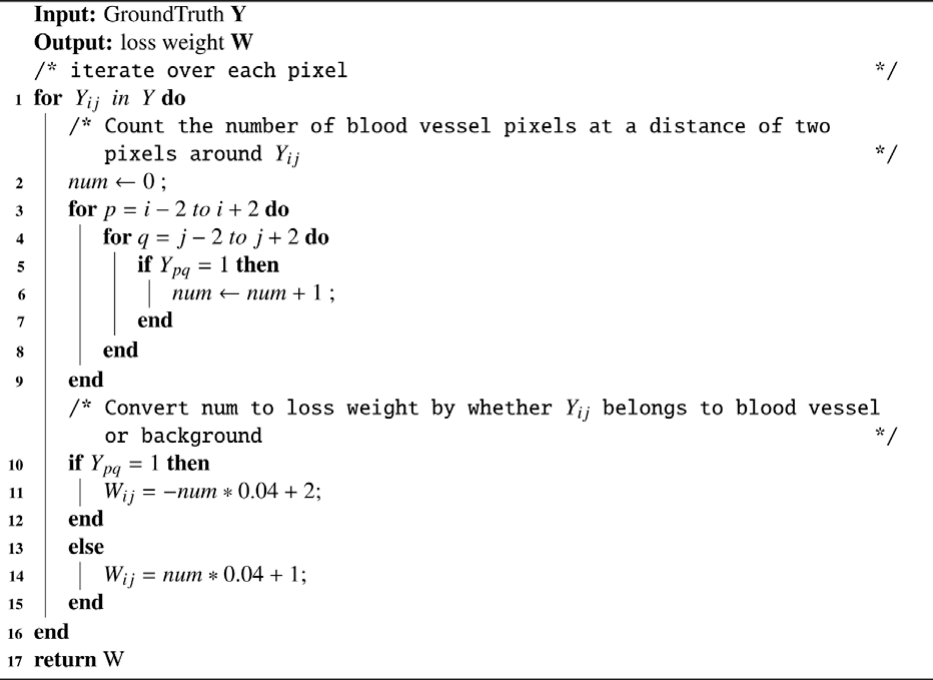

## 4 Experimental configuration

### 4.1 Dataset and augmentation

We evaluate the proposed method using two publicly available datasets (DRIVE^1^ and CHASE_DB1^2^). Specific information about these two databases is shown in Table 1. It should be noted that the original size of the two datasets is not suitable for our network, so we adjusted its size by zero padding around it, but the size was cropped to the initial size during evaluation. (See Table 1, Crop size). For the DRIVE dataset, the official data division is adopted, which means 20 training images were used for model training and 20 test images were used for performance evaluation. The CHASE_DB1 dataset has no official data division, so we follow the previous work (Alom et al., 2019; Wang et al., 2020b), using the first 20 images for model training, and the remaining 8 images for model evaluation. Furthermore, since the number of training images is limited to 20, some data augmentation methods are required. We use four data augmentation methods (see Table 1, Augmentation methods) for both datasets to generate randomly modified samples during the training process.

**TABLE 1:** The specific information of DRIVE and CHASE_DB1 datasets.

Datasets	DRIVE	CHASE_DB1
Form	A diabetic retinal disease screening study in the Netherlands	Comprehensive health study of 200 primary schools in the United Kingdom
Imaging equipment	Canon CR5 non-mydriatic 3CCD camera	NIDEK NM-200D Handy Fundus Camera
Total number	40	28
Train/Test number	20/20	20/8
Resolution (pixel)	584 × 565	999 × 960
Pad size	592 × 592	1008 × 1008
Augmentation methods	1) Random horizontal and vertical flip. 2) Random rotation. 3) color jittering.	

### 4.2 Evaluation metrics

To evaluate our method, we compare the segmentation results to the corresponding Ground Truths and classify the outcomes of each pixel comparison into True Positive (TP), False Positive (FP), False Negative (FN), and True Negative (TN). The model’s performance is then evaluated using sensitivity (SE), F1 score (F1), and accuracy (ACC), which are defined as:

$$SE=\frac{TP}{TP+FN}$$

$$SP=\frac{TN}{TN+FP}$$

$$ACC=\frac{TP+TN}{TP+TN+FP+FN}$$

$$F1=\frac{2TP}{2TP+FP+FN}$$

The closer the value of these evaluation metrics are to 1, the better the prediction. Furthermore, receiver operating characteristic (ROC) curves and the area under the ROC curve (AUC) were used to evaluate the performance of our model. The ROC curve was calculated as the variation of the TP and FP rate for different values of a changing threshold.

### 4.3 Implementation details

Our method is built on the PyTorch^3^ framework and all experiments were run on an NVIDIA RTX3090 with 24 GB of memory. We did not use any pre-trained models, and the entire training process was end-to-end without any post-processing. For the hyperparameter settings, the batch size was set to 2 for both datasets, and the network was optimized using an Adam (Kingma and Ba, 2014) optimizer with an initial learning rate of 1e-3. The total number of learning epochs was set to 200, and a learning rate decay by the factor 0.1 was performed at epochs 150 and 190. We used the best epoch of results for testing.

## 5 Results and discussions

### 5.1 Segmentation performance on two databases

**FIGURE 6:**
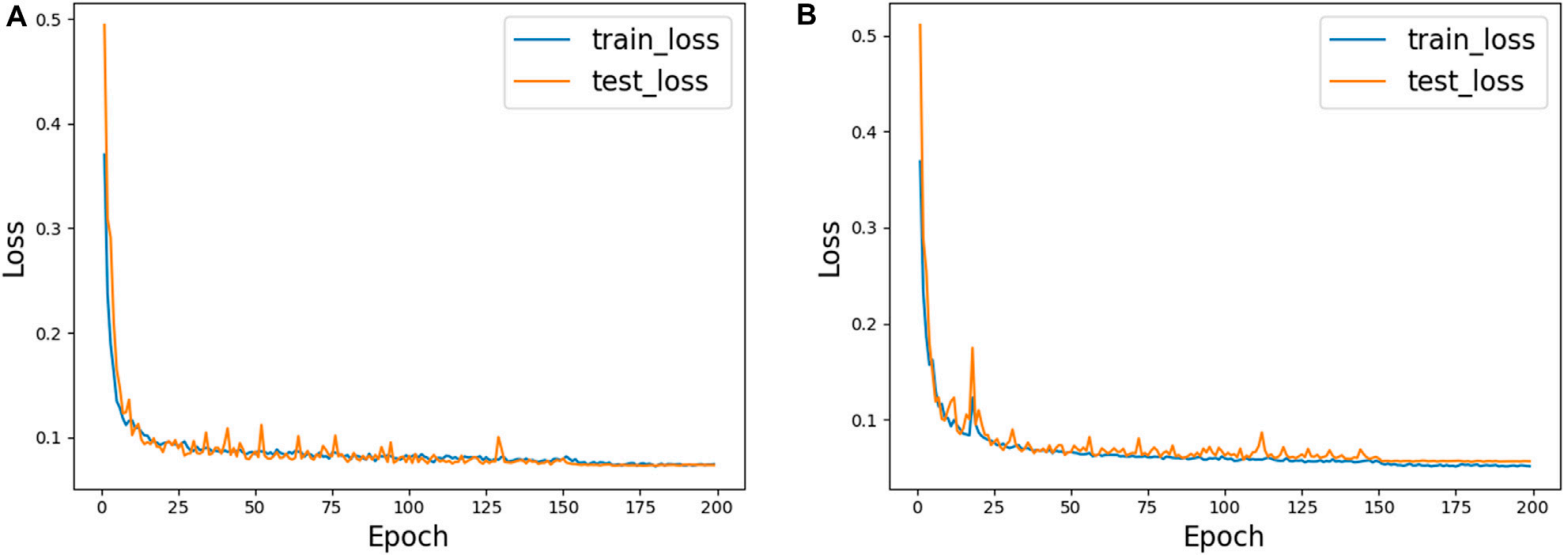
The training process of AGC-Net on two datasets. **(A)** DRIVE, **(B)** CHASE_DB1.

Figure 6 shows the training process of AGC-Ne in DRIVE and CHASE_DB1, where the blue line represents the loss change curve on the training set, and the orange line represents the loss change curve on the test set. We can observe that on the two data sets, the loss of AGC-Net on the training set and the test set can converge well, and the loss of the test set can be comparable to that of the training set. This shows that AGC-Net can adapt well to unseen data and has good generalization ability.

**FIGURE 7:**
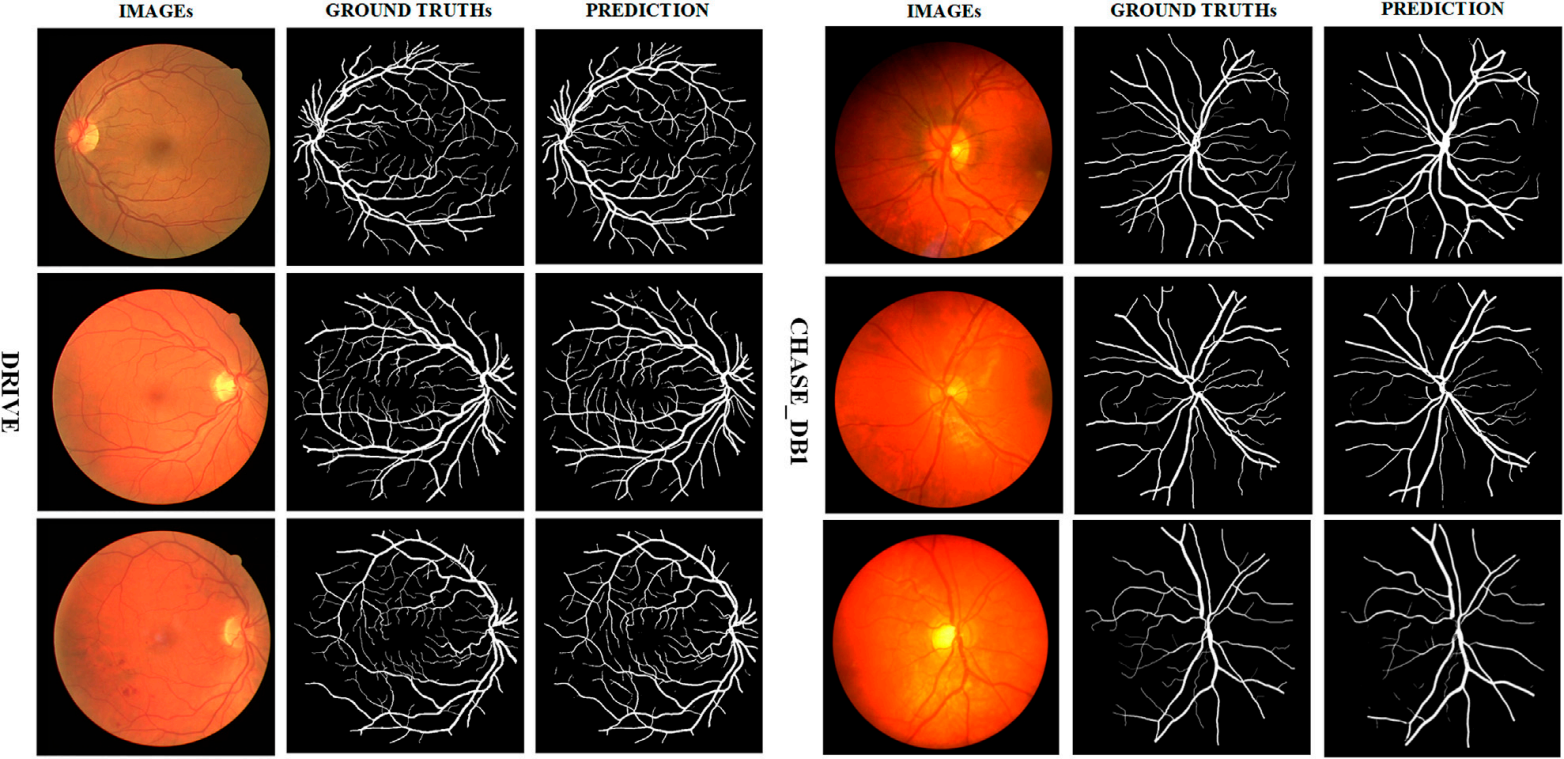
Example segmentation results on two datasets.

We present in Figure 7 some test images of the two datasets, their ground truth values, and the predictions generated by AGC-Net using these images. As can be seen from the figure, AGC-Net detects most retinal vessels on fundus images, including thin vessels with low contrast and thick vessels with over-illumination. Furthermore, the vessel thickness in our model predictions is consistent with the ground truth. Most of the spatial information of retinal vessels is preserved, such as vessel connectivity, bifurcations, and edges.

We also quantitatively evaluate AGC-Net on the two datasets separately. Table 2 presents the five metric values of our method on the two datasets. The table shows that on the two data sets, the SE, SP, ACC, F1, and AUC of AGC-Net can reach 0.8251/0.8499, 0.9844/0.9854, 0.9704/0.9767, 0.8301/0.8213 and 0.9881/0.9917 respectively. This demonstrates that our proposed AGC-Net model can generate accurate and meaningful retinal vessel segmentation, providing doctors valuable auxiliary diagnostic information in clinical practice.

**TABLE 2:** Performance of the proposed AGC-Net on DRIVE and CHASE_DB1 datasets.

Datasets	SE	SP	ACC	F1	AUC
DRIVE	0.8251	0.9844	0.9704	0.8301	0.9881
CHASE_DB1	0.8499	0.9854	0.9767	0.8213	0.9917

### 5.2 Ablation studies

As shown in Figure 2, AGC-Net can be regarded as a segmentation network composed of Cascade Design (CD), Auxiliary Supervision (AS), Inter-Stage Attention Module (ISAM), and Pixel-Importance-Balance Loss (PIBL). In this section, we conduct ablation studies to verify the effectiveness of these crucial components in AGC-Net and evaluate the impact of each component on the vessel segmentation results. We use Res-UNet (Xiao et al., 2018) with an initial channel number of 32 and only two downsampling stages as a baseline and gradually add the above crucial components. All experiments are performed with the same hyperparameter configuration. Table 3 shows the quantitative comparison of network configurations that incorporate different crucial components.

**TABLE 3:** Ablation studies with different network configurations.

Index	Baseline	CD	AS	ISAM	PIBL	SE (%)	SP (%)	ACC (%)	F1 (%)	AUC (%)
1	√					78.95	98.75	97.01	82.27	98.72
2	√	√				78.36	**98.78**	96.99	82.00	98.66
3	√	√	√			79.67	98.73	97.06	82.60	98.80
4	√	√	√	√		80.11	98.70	**97.07**	82.73	98.80
5	√	√	√		√	81.79	98.48	97.02	82.76	98.80
6	√	√	√	√	√	**82.51**	98.44	97.04	**83.01**	**98.81**

Baseline: Res-UNet; CD, Cascade Design; AS, Auxiliary Supervision; ISAM, Inter-Stage Attention Module; and PIBL, Pixel-Importance-Balance Loss. The value in bold is the highest value under that metric.

From Index 2 in Table 3, we can see that when we simply add another backbone to the baseline for cascading, SE, ACC, F1, and AUC all suffer a decline. This shows that adding the cascade design will bring optimization problems caused by increased network depth. That is, the gradient cannot be backpropagated well. As seen in Index 3, this problem can be solved after we add auxiliary supervision. Adding auxiliary supervision enables the cascaded design to further improve the baseline performance, among which SE, ACC, F1, and AUC are increased by 0.72%, 0.05%, 0.33%, and 0.08% compared with the baseline, respectively. Then, if we continue to add ISAM, by comparing index 3 and index 4 in the table, we find that SE, ACC and F1 continue to grow by 0.44%, 0.01%, and 0.13%, respectively. And when we don’t add ISAM but use PIB loss to train the network with index 3, by comparing index 3 and index 5 in the table, the SE and F1 of the network also improve, increasing by 2.81% and 0.16%, respectively, but SP and ACC have a slight drop. Finally, when we use both ISAM and PIB loss, SE, F1 and AUC reach the highest values of 82.51, 83.01, and 98.81 in Table 3, which are 3.56%, 0.74%, and 0.09% higher than the baseline, respectively. This is higher than the improvement obtained by adding ISAM or PIB loss alone, which shows that the two are compatible with each other and can promote performance improvement. But the SP reached the lowest value of 98.44%. The highest SE and the lowest SP reflect that our method further enhances the vessel extraction ability but inevitably introduces some false positives, which is acceptable (Moccia et al., 2018).

Furthermore, we plot some heatmaps generated using Grad-CAM (Selvaraju et al., 2017) in Supplementary Figure S1. In the heatmap, the redder the region’s color, the more the network pays attention to the feature of the region when predicting blood vessels. From Supplementary Figure S1, we can observe that the blood vessel area has been emphasized after adding ISAM to the network. This demonstrates that ISAM can promote fine-stage backbones in the network to focus on vascular regions and perform better refinement.

**FIGURE 8:**
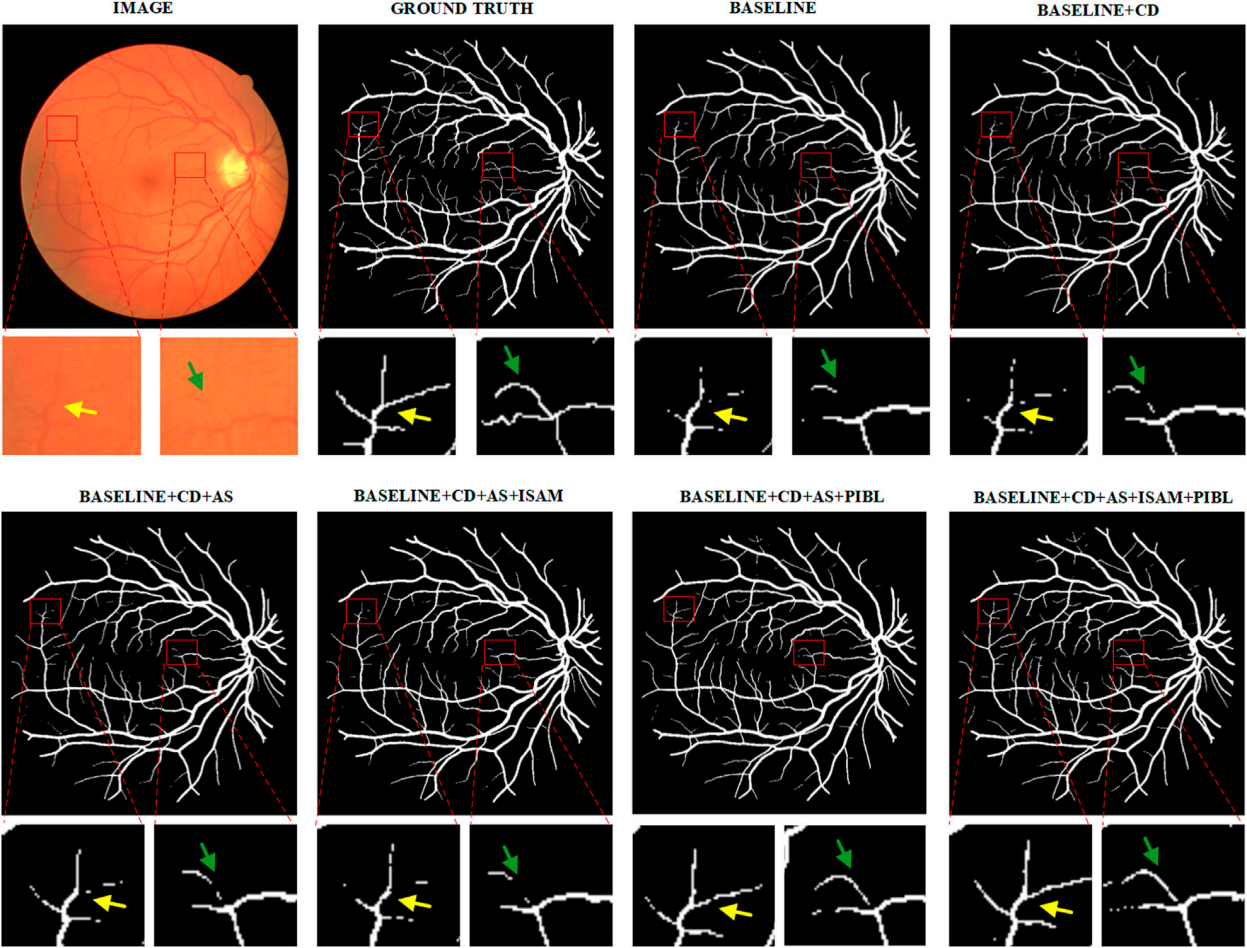
Example segmentation results for different network configurations on the DRIVE dataset. Baseline: Res-UNet; CD, Cascade Design; AS, Auxiliary Supervision; ISAM, Inter-Stage Attention Module, and PIBL, Pixel-Importance-Balance Loss.

We present example segmentation results of different network configurations in ablation studies in Figure 8 and further zoom in on some vessel regions under each image for qualitative comparison. As can be seen from the figure, the baseline after adding all the important components works best for the effect of vessel segmentation. This shows the necessity of every critical component.

### 5.3 Comparison with the state-of-the-art methods

**FIGURE 9:**
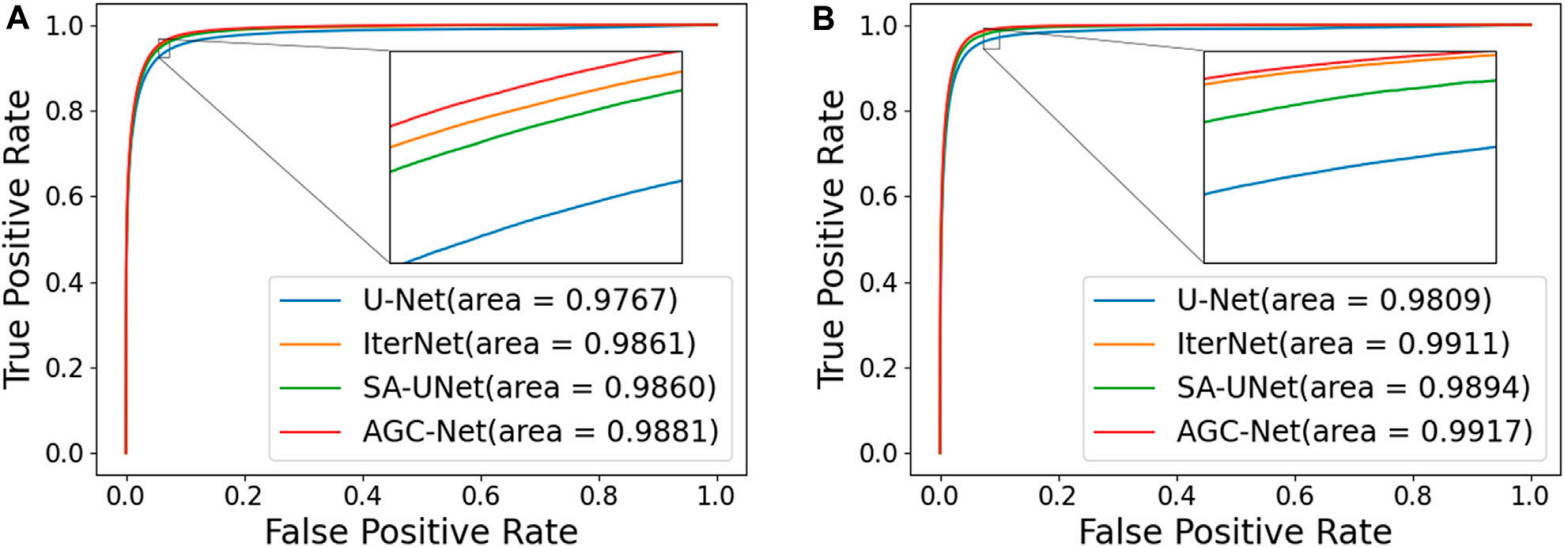
ROC curves and AUC value of different models on two Datasets. **(A)**: DRIVE, **(B)**: CHASE_DB1.

In this section, we compare the proposed method with some popular vessel segmentation methods, including U-Net (Ronneberger et al., 2015), IterNet (Li et al., 2020), and SA-UNet (Guo et al., 2021b). To test the results of these vessel segmentation methods, we used their public codes on the DRIVE and CHASE_DB1 datasets for training and evaluation. The Receiver Operating Characteristic (ROC) curves and AUC values of the four models on the two datasets are shown in Figure 9. The figure shows that compared with the suboptimal method, the AUC values obtained by AGC-Net have increased by 0.20% and 0.03% on the two data sets, respectively. Considering that these popular methods already have high performance (i.e., AUC values very close to 1.0), this improvement means that many vessel pixels can now be correctly classified.

In addition, we also compare some state-of-the-art methods in the literature, including R2UNet (Alom et al., 2019), DUNet (Jin et al., 2019), NFN+ (Wu et al., 2020), CAR-UNet (Guo et al., 2021a), RVSeg-Net (Wang et al., 2020b), SCS-Net (Wu et al., 2021), AG-Net (Zhang et al., 2019) and FR-UNet (Liu et al., 2022). Only the four methods in the previous paragraph come from our reproduced results, and the results of all other methods come from the corresponding papers. The results on the DRIVE dataset are listed in Table 4. Among all the compared methods, our method ranks second in ACC, F1 and AUC, and is very close to the first-ranked method (FR-UNet). Specifically, among these metrics, for the ACC value, our method achieves 0.9704, which is only 0.01% lower than FR-UNet. In addition, the AUC value and F1 value reached 0.9881 and 0.8301, respectively. In all comparison methods, same as FR-UNet, these two values exceed the values of 0.98 and 0.83. For the other two metrics SE and SP, the results obtained by AGC-Net are also comparable to other state-of-the-art methods. SE is usually interpreted as the model’s ability to correctly detect all vascular regions in retinal images. The SE obtained by our method can reach 0.8251, which is 1.05% lower than FR-UNet (0.8356). Nevertheless, this is still much higher than some other methods based on coarse-to-fine architectures [such as NFN+ (0.7796), CAR-UNet (0.8135) and IterNet (0.7921)]. The difference between AGC-Net and other methods based on coarse-to-fine architecture is that we use ISAM to enhance the container area of the intermediate output, which enables the backbone of the fine stage to better refine the container, resulting in higher SE value. SP is often interpreted as the localization ability of retinal vessel segmentation models. This ability refers to the ability to unerringly identify non-vascular regions as blood vessels. The SP of our method can reach 0.9844, which is 0.3% lower than the top-ranked IterNet (0.9874). We believe this is due to our method detecting more blood vessels, but inevitably introducing some false positives. Since the goal of the retinal vessel segmentation task is to detect as many vessels as possible, a relatively low SP is acceptable.

**TABLE 4:** Performance comparison of the DRIVE dataset.

Method	Year	SE	SP	ACC	F1	AUC
U-Net Ronneberger et al. (2015)	2015	0.7776	0.9867	0.9681	0.8108	0.9766
R2UNet Alom et al. (2019)	2018	0.7792	0.9813	0.9556	0.8171	0.9784
DUNet Jin et al. (2019)	2019	0.7963	0.9800	0.9566	0.8237	0.9802
AG-Net Zhang et al. (2019)	2019	0.8100	0.9848	0.9692	—	0.9856
IterNet Li et al. (2020)	2019	0.7921	**0.9874**	0.9699	0.8244	0.9861
NFN+ Wu et al. (2020)	2020	0.7796	0.9813	0.9582	0.8295	0.9830
RVSeg-Net Wang et al. (2020b)	2020	0.8107	0.9845	0.9681	—	0.9817
SCS-Net Wu et al. (2021)	2021	0.8289	0.9838	0.9697	—	0.9837
SA-UNet Guo et al. (2021b)	2021	0.8264	0.9823	0.9687	0.8224	0.9861
CAR-UNet Guo et al. (2021a)	2022	0.8135	0.9849	0.9699	—	0.9852
FR-UNet Liu et al. (2022)	2022	**0.8356**	0.9837	**0.9705**	**0.8316**	**0.9889**
AGC-Net (Our)	2023	0.8251	0.9844	0.9704	0.8301	0.9881

The value in bold is the highest value under that metric.

Table 5 shows the results of the different methods on the CHASE_DB1 dataset. It should be noted that the data partitioning methods of DUNet and NFN + are different from ours. Therefore, for the sake of fairness, we do not compare the results of these two methods. Unlike the case on the DRIVE dataset, on this dataset, our proposed AGC-Net exceeds FR-UNet and achieves 0.9767, 0.8213, and 0.9917 in ACC, F1 and AUC, respectively, which are the best results among all compared methods. Among these metrics, the AUC value best reflects the comprehensive performance of the model segmentation. In this experiment, our method reached 0.9917, which is very close to 1.0, which shows the good robustness of AGC-Net. For the ACC and F1 values, our method outperforms FR-UNet by 0.19% and 0.62%, respectively. FR-UNet is a segmentation framework that maintains full-resolution representation learning to retain more spatial information lost due to downsampling. However, the fundus images on the CHASE_DB1 dataset are already of high resolution (999 × 960) and have sufficient spatial information, which makes the advantage of FR-UNet on this dataset diminished. In addition, we can observe that some segmentation methods based on the same coarse-to-fine architecture also have higher ACC values than FR-UNet (0.9748), such as IterNet (0.9766) and CAR-UNet (0.9751). This suggests that for some high-resolution fundus images, a segmentation method based on the coarse-to-fine architecture may be a better choice. For the SE metric, AGC-Net achieves 0.8499, which ranks third among all compared methods and outperforms other methods based on coarse-to-fine architecture. This is because we designed a more reasonable loss function and used ISAM to promote the fine stage to achieve better refinement.

**TABLE 5:** Performance comparison on the CHASE_DB1 dataset.

Method	Year	SE	SP	ACC	F1	AUC
U-Net Ronneberger et al. (2015)	2015	0.7961	0.9863	0.9746	0.7974	0.9808
R2UNet Alom et al. (2019)	2018	0.7756	0.9820	0.9634	0.7928	0.9815
AG-Net Zhang et al. (2019)	2019	0.8186	0.9848	0.9743	—	0.9863
IterNet Li et al. (2020)	2019	0.8141	**0.9878**	0.9766	0.8165	0.9910
RVSeg-Net Wang et al. (2020b)	2020	0.8069	0.9836	0.9726	—	0.9833
SCS-Net Wu et al. (2021)	2021	0.8365	0.9839	0.9744	—	0.9867
SA-UNet Guo et al. (2021b)	2021	0.8651	0.9814	0.9740	0.8076	0.9893
CAR-UNet Guo et al. (2021a)	2022	0.8439	0.9839	0.9751	—	0.9898
FR-UNet Liu et al. (2022)	2023	**0.8798**	0.9814	0.9748	0.8151	0.9913
AGC-Net (Our)	2023	0.8499	0.9854	**0.9767**	**0.8213**	**0.9917**

The value in bold is the highest value under that metric.

Through qualitative comparisons on the two datasets, we find that both AGC-Net can guarantee the improvement of comprehensive segmentation performance and maintain a high SE without introducing too many false positives. Therefore, compared to other methods, we believe that AGC-Net can better cope with the vessel segmentation task.

**FIGURE 10:**
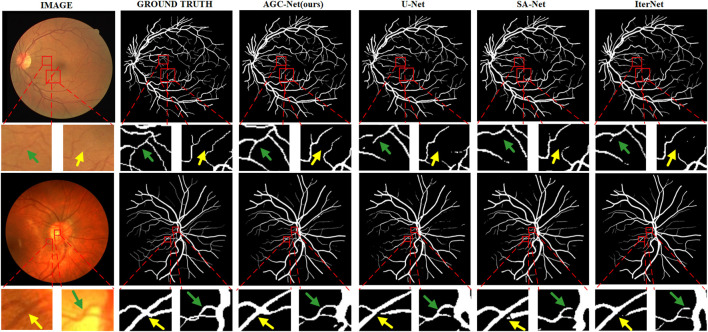
Example segmentation results of different models on two datasets. From left to right are image, ground truth, prediction result of AGC-Net, prediction result of U-Net, prediction result of SA-UNet, and prediction result of IterNet. From top to bottom are the DRIVE dataset and the CHASE_DB1 dataset.

Especially when we compare the segmentation results of different methods in Figure 10, the advantages of AGC-Net are more prominent. It can be seen from the figure that the blood vessel segmentation results obtained by other methods lack sufficient semantic information, and the blood vessels are broken. The segmentation result of our method is closer to the ground truth, as it identifies some blood vessels that other methods cannot identify, including over-illuminated blood vessels and low-contrast thin blood vessels, and the connectivity of blood vessels is better. There are three reasons for the superior performance of AGC-Net on visual effects: First, the fine stage in the AGC-Net framework gives those misclassified vessel pixels a chance to be corrected. Second, ISAM improves the degree of attention of the fine-stage backbone to the vessel region, which achieves a better refinement effect. Third, the PIB loss scales the loss weights per pixel so that certain key pixels contribute more to the gradient. The advantages of AGC-Net in qualitative comparison with other methods can provide doctors or experts with more useful vascular information in practical applications. This can facilitate early detection and treatment of eye diseases.

For the problem of imbalance between foreground pixels and background pixels in fundus images, PIB Loss is very effective. We recommend that other researchers use PIB Loss to improve performance when training blood vessel segmentation models. If other researchers are designing a segmentation model based on a coarse-to-fine architecture, we suggest using ISAM to improve the refinement effect of the fine stage.

### 5.4 Limitations

Although our method performs very well compared to other methods, several limitations exist. First, the proposal of PIB Loss can significantly alleviate the problem of an unbalanced ratio of foreground pixels and background pixels in fundus images. However, due to the selection of pixel distance (we fixed it as a box with a distance of 2 pixels in the method) coupled with the proportional function, PIB Loss still needs to be flexible enough. This limit exploring the effect of pixel distances of 3 or more pixels on experimental results. In future work, we plan to decouple the pixel distance and proportional function of PIB Loss and explore the impact of more pixel distances on experiments. Second, although our method has segmented more blood vessels than other methods, there are still breaks or unrecognized phenomena for some extremely small blood vessels. This is attributed to the amount of training data being too small (usually only around 20 capacity), which leads to poor generalization on these extremely small blood vessels. We plan to explore more effective data augmentation techniques in future work.

## 6 Conclusion

Our paper presents a novel method for segmenting retinal vessels, which are essential for diagnosing and treating eye diseases. The proposed method designs a coarse-to-fine network with a two-stage strategy: the first stage generates a rough vessel prediction map, and the second stage corrects the misclassified pixels in this map. The coarse-to-fine network uses a novel inter-stage attention module to adjust the importance of vessel regions in the intermediate output for better refinement. In addition, we design a novel PIB loss for network training to address the problem of pixel ratio imbalance in fundus images. PIB avoids the gradient being dominated by many background pixels by scaling the loss weight of each pixel, which is of great help to improve the blood vessel segmentation effect. We evaluated our method on two public datasets and found that it outperformed several state-of-the-art methods with high performance.

## Data Availability

The original contributions presented in the study are included in the article/Supplementary Material, further inquiries can be directed to the corresponding authors.

## Footnotes

1 DRIVE: http://www.isi.uu.nl/Research/Databases/DRIVE/

2 CHASE_DB1: https://blogs.kingston.ac.uk/retinal/chasedb1/

3
https://pytorch.org/
