# Supplementary figures and images for: Attention-guided cascaded network with pixel-importance-balance loss for retinal vessel segmentation
Source: Front Cell Dev Biol. 2023 May 9;11:1196191. doi: 10.3389/fcell.2023.1196191 (PMC10203622; doi:10.3389/fcell.2023.1196191)

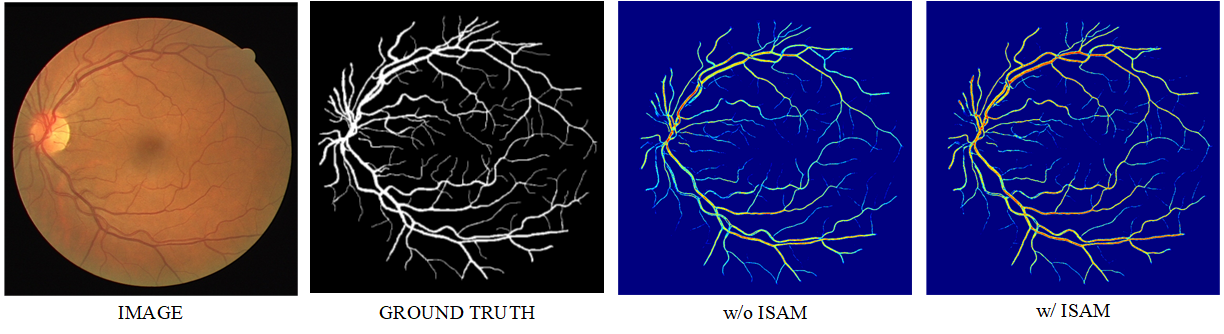

Supplement: Supplementary file 1 [file Image1.tif]
